# *Streptomyces thermovulgaris* Bacteremia in Crohn's Disease Patient

**DOI:** 10.3201/eid1010.040300

**Published:** 2004-10

**Authors:** Miquel Bart Ekkelenkamp, Wilma de Jong, Willem Hustinx, Steven Thijsen

**Affiliations:** * Utrecht University, Utrecht, the Netherlands;; † Diakonessenhuis, Utrecht, the Netherlands

**Keywords:** letter, Streptomyces, bacteremia, Crohn’s disease, immunosuppression

**To the Editor:** Invasive infections with *Streptomyces* spp. are rare; in reference to two cases reported in Emerging Infectious Diseases (*1**,**2*), we describe here the first documented case of bacteremia with *Streptomyces thermovulgaris*. An 81-year-old woman was admitted to the emergency room of Diakonessenhuis, Utrecht, the Netherlands, with severe abdominal pain in the right lower quadrant and feculent vomitus. The patient had a history of Crohn's disease, for which she had undergone resection of the ileum and cecum, and was receiving high-dose corticosteroid therapy (prednisone 25 mg daily). The patient also had steroid-induced osteoporosis and an internal pacemaker. On admission, the patient had a temperature of 35.6°C, a leukocyte count of 8.4 x 10^9^/L with 30% bandforms, and a C-reactive protein level of 18 mg/L. Moreover, the patient had severe metabolic acidosis and was hemodynamically unstable, suggesting septic shock subsequent to a presumed bowel perforation.

Blood samples were drawn and cultured, and empiric treatment was initiated with ceftriaxone and metronidazole. Exploratory laparotomy showed colonic inflammation. The patient was transferred to the intensive care unit postoperatively. On day 9 of hospitalization, the blood culture taken on day 1 before antimicrobial drug was started, turned positive in the automatic blood culture system. Gram staining showed gram-positive nocardioform rods, and subculture on tryptic soy agar with 7% sheep blood yielded polymorphous colonies that sunk into the agar and showed filamentous growth.

Despite intensive antimicrobial and supportive therapy, sepsis progressed into multiple organ failure with severe neuropathy. On day 25 of hospitalization, the patient died. Autopsy demonstrated no possible infective focus except severe inflammation of the colon and distal ileum. Permission for cerebral section was not granted. The blood isolate showed strictly aerobic growth at 37°C and 50°C with β-hemolysis and was positive for catalase, caseinase, gelatinase, and nitrate reduction; the isolate was negative for oxidase, urease, and esculin hydrolysis and was nonmotile at 37°C. The isolate was sent to the National Institute of Public Health, Bilthoven, the Netherlands, for identification. Biochemical analysis, fatty acid analysis, and 16S rRNA typing identified the strain as *S. thermovulgaris*. The strain was susceptible to ceftriaxone (MIC 0.32 µg/mL) by Etest (BA Biodisk, Solna, Sweden). Further susceptibility testing performed by agar diffusion showed that the organism was also susceptible to amoxicillin, vancomycin, a combination of trimethoprim and sulfamethoxazole, and erythromycin.

Bacteremia produced by a strain of *S. thermovulgaris*, as we report here, is the first documented case of isolation of this microorganism from human material. The streptomycetes are classified as a separate genus within the aerobic actinomycetes and are most well known for the many antimicrobial substances isolated from the approximately 600 different species (*3*). Streptomycetes, aerobic, spore-forming, gram-positive bacteria with filamentous growth, are ubiquitous in soil and can cause mycetomas (*4*). Streptomycetes are widely distributed in terrestrial and aquatic habitats with soil, fodder, and compost as their primary reservoirs. The amount of actinomycetes (the taxonomic group to which the streptomycetes belong) in soil is estimated to be 10^7^–10^8^ microorganisms per gram (*5*), and in total biomass equal to that of all other bacteria together and slightly less than that of fungi. *S. thermovulgaris* belongs to the thermophilic streptomycetes, which do not grow at temperatures <37°C and are believed to affect biodegradation of organic waste products at higher temperatures (*6*). The *S. thermovulgaris* strains in the American Type Culture Collection have been isolated from manure, manured soil, and compost.

*S. anulatus*, *S. somaliensis*, and *S. paraguayensis* are the species that have been implicated most frequently as causing human disease, but streptomycetes such as *S. albus* (*7*), *S. coelicolor*, *S. lavendulae*, *S. rimosus*, *S. bikiniensis*, and *S. violaceoruber* have also been implicated (*2*). Invasive infections caused by streptomycetes are very rare, and few cases of bacteremia have been reported (*1**,**2**,**8*).

Reported clinical isolates are often associated with decreased immunity, as in the case of *S. bikiniensis* from a patient with osteosarcoma (*2*), and in the case of *Streptomyces* spp. from patients with HIV (*9*). Our patient had severe immunosuppression as a result of intensive steroid treatment for therapy-resistant Crohn's disease. Autopsy identified no possible focus of infection other than the patient's intestines, which were severely inflamed with massive ulceration. Cultures taken at autopsy were negative.

Because of the large numbers of streptomycetes in the agricultural environment, eating contaminated food probably occurs frequently; however, in healthy people it will not lead to invasive infection. In our patient, the heavily inflamed, ulcerated gut likely enhanced the opportunity for infection from the intestines.

Because it is a soil bacterium, *Streptomyces* spp. would not likely contaminate a hospital environment. Moreover, studies have never shown *Streptomyces* spp. as contaminants (*10*); therefore, any clinical isolate should be considered potentially relevant. After antibiotic therapy was initiated, repeated blood cultures showed no persisting *Streptomyces* bacteremia, which can be explained by the strain's susceptibility to the antimicrobial agents that were given.

In conclusion, we have described the first documented case of *S. thermovulgaris* bacteremia. Immunocompromised patients are susceptible to colonization and infection with a broad range of both common and uncommon pathogens. Although the clinical value of positive blood cultures with less common pathogens such as streptomycetes must always be carefully weighed, they may not simply be discarded as contaminants.
